# Real-time MRI feedback of cavitation ablation therapy (histotripsy)

**DOI:** 10.1186/2050-5736-3-S1-O89

**Published:** 2015-06-30

**Authors:** Steven Allen, Timothy Hall

**Affiliations:** 1 University of Michigan, Ann Arbor, Michigan, United States

## Background/introduction

Histotripsy ablation of liver tumors is a non-invasive surgery that uses high-intensity acoustic pulses to control an inertial cavitation cloud. Repeated exposure to the cavitation cloud renders a target tissue into a homogenous slurry which is then gradually absorbed by the body. Like other non-invasive surgeries, histotripsy requires a feedback system that can estimate therapy location and dose in real-time. Because the time-average power output of histotripsy is very small, the treatment region does not express a significant rise in temperature, making MRI thermometry an ineffective method to actively monitor therapy. Previous work has shown that MRI pulse sequences can be made sensitive to the chaotic water flow present during inertial cavitation. This is done by synchronizing the timing of the histotripsy pulses with the timing of the sequence’s gradient waveforms. Incoherent water flow caused by cavitation attenuates the MR signal through a process similar to that used in diffusion-weighted MRI. It was shown that these sequences can give localized contrast specific to histotripsy bubble clouds. However, the MR images could only be acquired in a piecemeal fashion over several minutes such that a single image represented the influence of many cavitation events. Here, we introduce a single-shot MR acquisition sequence that is able to rapidly acquire a complete MR image and remain sensitive to histotripsy cavitation. This is done by synchronizing each histotripsy pulse with incoherent motion-weighting gradients placed just before the readout portion of the sequence. When repeated at the same rate as the histotripsy pulses, the resulting MR images can give feedback on the location of every cavitation cloud applied to the target tissue.

## Methods

**Figure 1:**
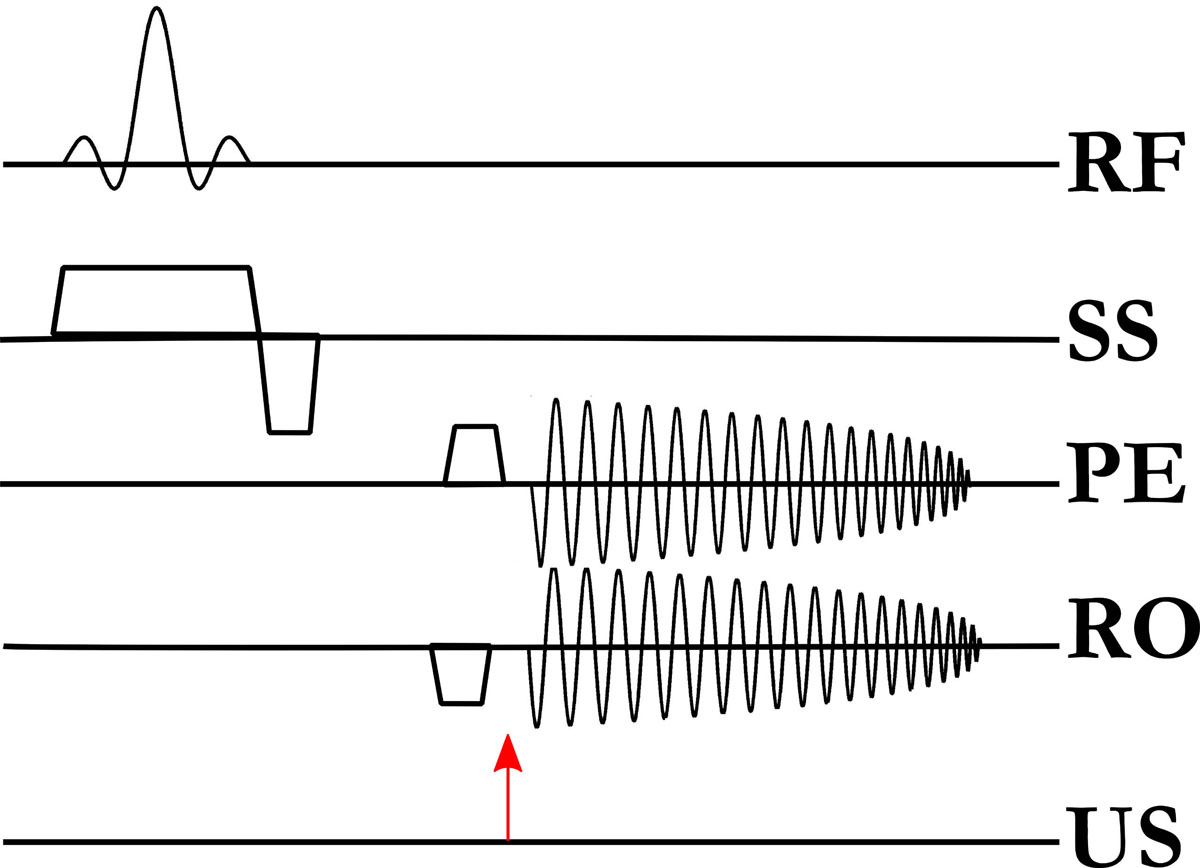
Cavitation sensitive, single-shot pulse sequence. A histotripsy pulse (arrow) is placed in between motion encoding gradients and the readout gradients. RF = radio frequency, SS = slice select, PE = phase encode, RO = readout, US = ultrasound.

An MR-compatible transducer was placed in the bore of a 7T MRI scanner and coupled to a water bath and a 2 cm surface RF coil. A cavitation sensitive, single-shot, spiral readout sequence was used to repeatedly image a plane transecting the focus of the transducer. Acoustic pulses from the transducer were triggered to fire between the incoherent-motion sensitizing gradients of the sequence and the readout gradients. See figure (1). Control images were also acquired with the sensitizing gradients disabled.

## Results and conclusions

**Figure 2:**
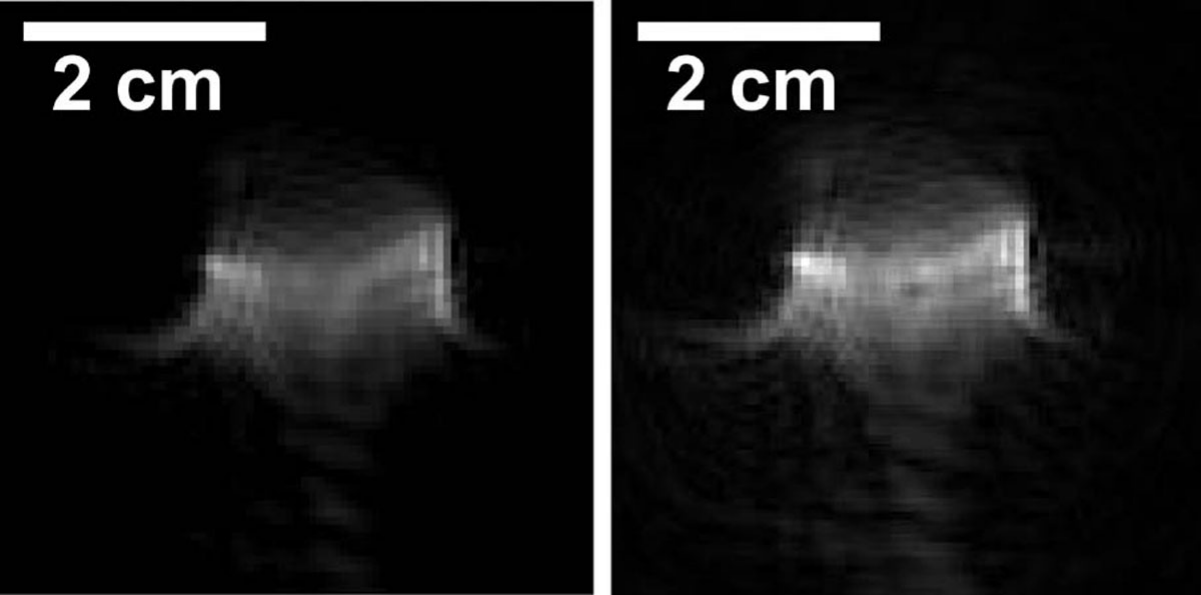
Single shot, cavitation sensitive image of the transducer focus with the weighting gradients turned off (left). When the weighting gradients are turned on, a small region of attenuation appears at the transducer focus (right).

An example control image and an example cavitation weighted image are shown in figure 2. In the figure, the surface coil is centered about the transducer focus while the transducer itself and the majority of the water bath lie outside the field of view. The acoustic pulses propagate from bottom to top of the image. The cavitation weighted image displays a small region of signal attenuation at the transducer focus which is not present in the control image. Each image is acquired over the course of 30 ms and repeated every 400 ms, resulting in localization information for every cavitation pulse applied to the target.

